# Incidence of deep venous thrombosis in patients with hemophilia undergoing bilateral simultaneous total knee arthroplasty: a retrospective cohort study

**DOI:** 10.1186/s12891-024-07404-2

**Published:** 2024-04-24

**Authors:** Qian Zhang, Lingying Zhao, Nicoletta Riva, Ziqiang Yu, Miao Jiang, Alexander Gatt, Jiong Jiong Guo

**Affiliations:** 1 Department of Orthopedics and Sports Medicine, The First Affiliated Hospital of Soochow University, Suzhou, PR China; 2 Department of Hematology, National Clinical Research Center for Hematologic Disease, The First Affiliated Hospital of Soochow University, Suzhou, PR China; 3 Jiangsu Institute of Hematology, Key Laboratory of Thrombosis and Hemostasis of Ministry of Health of PR China, Suzhou, PR China; 4 Department of Pathology, Faculty of Medicine and Surgery, University of Malta, Msida, Malta; 5 Department of Haematology, Mater Dei Hospital, Msida, Malta; 6 China-Europe Sports Medicine Belt-and-Road Joint Laboratory of Ministry of Education of PRC, Suzhou, PR China

**Keywords:** Deep venous thrombosis, Hemophilia, Bilateral simultaneous total knee arthroplasty, Ultrasonography

## Abstract

**Background:**

Hemophilic arthropathy usually affects the knees bilaterally. In order to reduce costs and improve rehabilitation, bilateral simultaneous total knee arthroplasty (TKA) can be performed. However, pharmacological prophylaxis for deep venous thrombosis (DVT) remains controversial in patients with severe hemophilia. The purpose of this study was to establish the incidence of DVT in severe hemophilia A patients undergoing bilateral simultaneous TKA without pharmacological thromboprophylaxis.

**Methods:**

Consecutive patients with severe hemophilia A undergoing bilateral simultaneous TKA at a single center between January 2015 and December 2020 were retrospectively reviewed. All patients received a modified coagulation factor substitution regimen. Tranexamic acid (TXA) was used for hemostasis in all patients during surgery. All patients followed a standardized postoperative protocol with routine mechanical thromboprophylaxis, and none received anticoagulation. D-dimer was measured preoperatively, on the day of the operation and on postoperative days 1, 7 and 14. Ultrasound (US) of the lower extremities was performed before (within 3 days of hospitalization) and after surgery (days 3 and 14) to detect asymptomatic DVT. Patients were followed up until 2 years after surgery for the development of symptomatic DVT or pulmonary embolism (PE).

**Results:**

38 male patients with severe hemophilia A underwent 76 simultaneous TKAs. Mean (± standard deviation) age at the time of operation was 41.7 (± 17.1) years. Overall, 47.3% of patients had D-dimer concentrations above the threshold 10 µg/mL on day 7 and 39.5% on day 14. However, none of the patients had DVT detected on postoperative US, nor developed symptomatic DVT or PE during the 2-year follow-up.

**Conclusions:**

The risk of DVT in patients with severe hemophilia A after bilateral simultaneous TKA is relatively low, and routine pharmacological thromboprophylaxis may not be needed.

## Background

Hemophilia is an inherited bleeding disorder characterized by a deficiency in coagulation factor VIII (hemophilia A) or factor IX (hemophilia B). Recurrent joint bleeding (hemarthroses) can lead to hemophilic arthropathy, which usually affects the knees bilaterally [1, 2]. Total knee arthroplasty (TKA) is the most efficient, radical intervention for end-stage hemophilic knee osteoarthritis [3–6]. In order to reduce costs, improve rehabilitation, and avoid unnecessary medical therapy that could potentially lead to postoperative complications, bilateral simultaneous TKA can be performed in patients with severe hemophilia A [7–9].

Deep venous thrombosis (DVT) and pulmonary embolism (PE), collectively known as venous thromboembolism (VTE), are very common complications after TKA [10]. In non-hemophilic patients, 40–60% rates of asymptomatic DVT after TKA have been described in previous studies [11]. However, patients with hemophilia have a low risk of thromboembolic complications due to their coagulation factor deficiency [12]. According to the DVT prophylaxis guidelines, pharmacological thromboprophylaxis is not generally recommended in patients with these disorders because hemophilia is a risk factor for bleeding [13].

Prophylactic coagulation factor replacement therapy is recomended to use perioperatively to correct the hemostatic defect and reduce the risk of intraoperative bleeding [14, 15]. This therapy theoretically restores coagulation back to normal and potentially increases the risk of DVT in hemophiliac patients [16]. However, there are few data concerning the risk of DVT associated with simultaneous bilateral TKA in patients with hemophilia [17]. Consequently, pharmacological thromboprophylaxis is controversial for hemophilic patients who undergo total joint replacements especially simultaneous bilateral TKA [7, 12].

Given the low risk of DVT in patients with severe hemophilia, we hypothesized routine pharmacological thromboprophylaxis after bilateral simultaneous TKA is unnecessary. Thus, the purpose of this retrospective study was to establish the incidence of DVT in patients with severe hemophilia A undergoing bilateral simultaneous TKA without pharmacological thromboprophylaxis, as per normal practice within our institution.

## Materials and methods

### Study population

Consecutive patients with hemophilia A undergoing simultaneous TKA at a single center (*blind the name*) between January 2015 and December 2020 were retrospectively reviewed. The study protocol of this research was authorized by the local ethics committee (NO. 2021 − 450) and each patient signed an informed consent form. The inclusion criteria included: (1) patients diagnosed with severe hemophilia A; (2) patients undergoing bilateral simultaneous TKA successfully; (3) patients without active bleeding event before surgery. We excluded subjects with: (1) a history of VTE in the previous 5 years; (2) any previous anticoagulant treatment use for any indication; (3) severe ipsilateral or contralateral hip or ankle hemophilic arthropathy; (4) a history of other major surgeries within 2 years follow-up.

The following information was collected at enrolment: age, sex, body mass index (BMI), history of VTE, infection by human immunodeficiency virus, severity of hemophilia.

Criteria for classification of the severity of hemophilia followed the recommendations of the Scientific Standardization Committee of the International Society on Thrombosis and Haemostasis: severe hemophilia if factor VIII plasma levels < 1% [18].

### Surgical procedure of TKA and rehabilitation

A single surgical team carried out identical TKAs on both knees consecutively, under general anesthesia. A tourniquet was applied in all cases before the operation was performed and the pressure was set at 350 mmHg. The tourniquet was deflated after cementation but before closure. Both lower limbs were prepared and draped simultaneously. An extensive synovium removal was performed after a standard midline incision and a medial parapatellar arthrotomy. When exposure was limited, a quadriceps snip was performed for patients with severe limitation of flexion. A posterior stabilized model (Attune Primary Knee System, DePuy Synthes, USA) was cemented (gentamicin bone cement) as the prosthesis. We performed patella surface smoothing, and osteophyte removal for all the cases. Tranexamic acid (TXA) 1 g was administered intravenously 15 min before incision and 2 g were injected intraarticularly into all knees. TXA was also administered in the surgical area to the subcutaneous tissues, periarticular ligaments, and capsule after closure of arthrotomy. After the incision on one side was closed, the surgical area was sterilized and draped again. Then, an identical procedure on the other side was started. Suction drains were placed in both incisions, and they were removed 2–3 days after surgery when the drainage were less than 50 ml.

In accordance with institutional protocols, all patients underwent a standard rehabilitation program, utilizing auxiliary motion devices to mobilize the knee and encouraging early full-weight bearing. Range-of-motion exercises were started on postoperative day 2, knee exercises with continuous passive motion were started on day 3, walking with parallel bars was started on day 7 and walking with crutches was started on day 14 [16, 19].

### Study procedures: factor replacement and thromboprophylaxis

In our study, none of the patients had an active bleeding event before surgery. Therefore, coagulation factor replacement therapy was used for perioperative prophylactic hemostasis as recommended [14, 15]. Clotting factor was infused according to our modified hemostatic therapy plan for patients with hemophilia A. During preoperative pharmacokinetic testing, we determined the program of perioperative coagulation factor replacement by measuring plasma concentrations of FVIII. The target concentrations of FVIII were set at approximately 100% on the day of operation, at about 80% during the first 3 postoperative days, and at 60% during the fourth to seventh postoperative days. We administered an infusion of recombinant FVIII in a single bolus 1 h before surgery, followed by another bolus at the same dose, if the operation lasted more than 6 h. We started with an initial dose of 50 IU/kg on each patient, which was subsequently adjusted based on the target mentioned above. On postoperative days 1, 3, 5 and 7, we measured plasma coagulation FVIII levels, presence of inhibitors, and hemoglobin levels. We maintained the substitution of coagulation FVIII at a concentration of approximately 20% until suture removal, which was performed 2 weeks postoperatively.

None of the patients received anticoagulants as thromboprophylaxis. We applied both intermittent pneumatic compression devices and graduated compression stockings as mechanical prophylaxis methods to all patients.

### Study outcomes

Ultrasound (US) of the lower extremities was performed by a skilled US technologist before surgery (within 3 days since hospitalization) and after surgery (days 3 and 14), to detect asymptomatic DVT (using the US system Aplio 400 Platinum; Canon Medical Systems). A compression US with Color Doppler imaging was performed to assess both proximal and distal veins (femoral, popliteal, peroneal, posterior tibial, soleal veins, and great saphenous vein). D-dimer (latex enhanced immunoturbidimetric assay of D-dimer, on the analyzer ADVIA 2400, Siemens AG) was measured preoperatively (within 3 days of hospitalization), on the day of the operation and on postoperative days 1, 7 and 14 [12]. The manufacturer’s cut-off for this D-dimer is 2 µg/mL; however, since a previous study suggested that a cut-off D-dimer value of 10 µg/mL on postoperative day 7 might suggest DVT, both thresholds were analyzed [20].

Information was also collected on the development of complications, such as hemarthrosis, severe bleeding (defined as hemoglobin < 80 g/L) and infections during hospitalization.

Patients were followed up on an outpatient or telephone basis to determine the occurrence of symptomatic VTE (i.e., DVT or PE) at 3, 6, 12 and 24 months postoperatively.

### Statistical analysis

The data analysis was performed using SPSS 26.0 software (SPSS Inc., Chicago, Illinois, USA). Descriptive statistics was used: continuous variables are presented as mean (± standard deviation [SD]); categorical variables are presented as count and percentages; the numbers were reported to one decimal place. Given the small number of patients enrolled and the very low incidence of VTE, inferential statistical analysis was not used.

## Results

### Patient characteristics

Overall, 38 patients with hemophilia A who underwent 76 simultaneous TKAs were reviewed. The patients’ demographic data and characteristics are shown in Table 1.

**Table 1:** Baseline patients’ characteristics

Characteristic	N. of patients (*N* = 38)
Male sex, n (%)	38 (100%)
Age (years), mean ± SD (range)	41.7 ± 17.1 (19–60)
BMI (kg/m^2^), mean ± SD (range)	20.3 ± 4.1 (17.1–27.2)
History of VTE, n (%)	0 (%)
HIV, n (%)	6 (15.7%)
Severity of hemophilia, n (%)	Severe 38 (100%)

BMI: body-mass index; VTE: venous thromboembolism; HIV: human immunodeficiency virus; SD: standard deviation

*Note*. Values are expressed as mean? standard deviation and min-max range unless otherwise indicated

### Peri-operative management

**Fig. 1:**
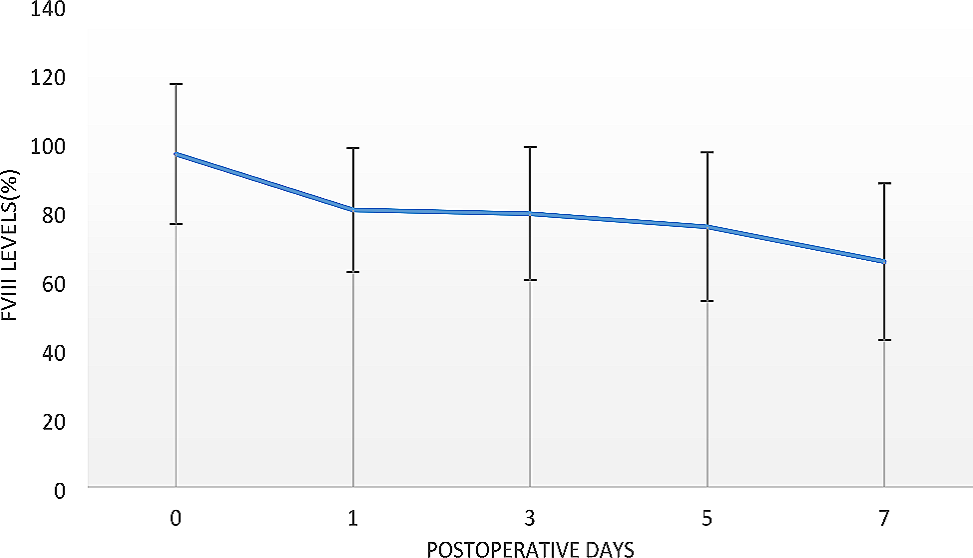
Time course of plasma factor VIII levels

The mean surgical time was 203.1 ± 40.7 min. The mean tourniquet time for each knee was 53.5 ± 6.7 min. All patients used mechanical thromboprophylaxis from the immediate postoperative period until discharge. The mean duration of hospital stay was 18.1 ± 5.4 days. Plasma mean FVIII level during the hospital stay is shown in Fig. 1. The mean FVIII consumption of each patient for operation and in hospital rehabilitation was 61,995 ± 20,039 IU.

### Study outcomes and complications

DVT was not found in either pre- nor post-operative US examinations. A review of the case notes of all patients found that none developed clinical signs and symptoms of VTE up to 24 months after surgery. At 24 months follow-up, 29 patients (76.3%) were assessed in the outpatient department, and 9 patients (23.7%) were contacted by telephone.

**Fig. 2:**
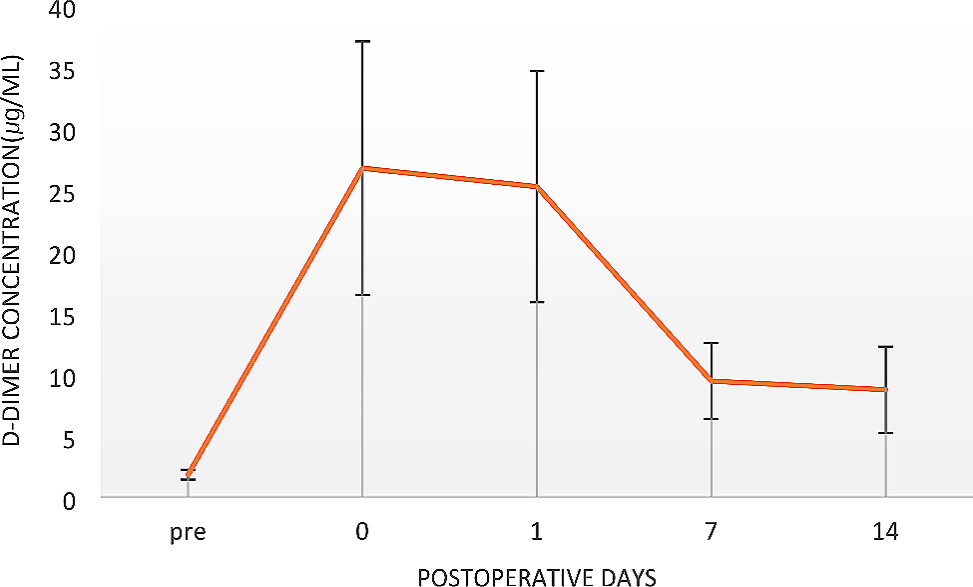
Time course of D-dimer concentration

Mean D-dimer concentrations were 1.8 ± 0.4 µg/mL preoperatively, 25.2 ± 9.4 µg/mL on postoperative day 1, 9.4 ± 3.1 µg/mL on postoperative day 7 and 8.7 ± 3.4 µg/mL postoperative day 14 (Fig. 2). Table 2 shows the number of patients that had D-dimer concentrations above the thresholds (> 2 µg/ml and > 10.0 µg/ml) at different time points.

**Table 2:** Patients’ outcomes

Outcome	N. of patients (*N* = 38)	
Severe bleeding (hemoglobin < 80 g/L), n (%)	0 (0%)	
Hemarthrosis, n (%)	2(5.2%)	
Infection, n (%)	0 (0%)	
Development of factor inhibitors, n (%)	0 (0%)	
Asymptomatic DVT during hospital stay, n (%)	0 (0%)	
Symptomatic VTE during follow-up, n (%)	0 (0%)	

DVT: deep vein thrombosis; VTE: venous thromboembolism

One patient developed hemarthrosis of the left knee 5 days after surgery. The hematoma was treated by arthrocentesis and 200 ml effusion were drained. Another patient developed hemarthrosis of the left knee on day 7 postoperatively. The patient was treated by conservative treatment including elevation of limb and ice compress. None of the patients developed factor inhibitors (Table 3). There were no other severe complications reported such as periprosthetic fractures or loosening of components in the 24 months follow-up.

**Table 3:** Patients with D-dimer concentrations above the thresholds

Time points	Threshold 2 µg/mL N. of patients (*N* = 38)	Threshold 10 µg/mL N. of patients (*N* = 38)
Pre-operation, n (%)	13 (34.2%)	0 (0%)
Day of operation, n (%)	38 (100%)	38 (100%)
Day 1, n (%)	38 (100%)	38 (100%)
Day 7, n (%)	38 (100%)	18 (47.3%)
Day 14, n (%)	38 (100%)	15 (39.5%)

## Discussion

The main finding of our study is that the risk of DVT in patients with severe hemophilia A treated with bilateral simultaneous TKA is relatively low. Although no pharmacological thromboprophylaxis was applied, asymptomatic DVT was not detected on postoperative US and none of the patients developed symptomatic VTE up to 2 years after surgery. In this group of patients, routine pharmacological thromboprophylaxis measures may not be necessary.

In accordance with the 2018 guidelines from the American Society of Hematology, the diagnosis of DVT should involve both D-dimer and US [21]. However, since D-dimer normally rises above the manufacturers’ threshold after surgery, its utility in the post-operative period has been questioned [22]. A previous study suggested a threshold value for D-dimer > 10.0 µg/ml at 7 days after TKA as possibly indicative of DVT [20]. In our study, though 18 patients had D-dimer values above this threshold on postoperative day 7 and 15 patients on day 14 (Table 3), no DVT was detected on these patients on US. In addition, during a two-year follow-up, no patient reported symptomatic VTE events. These findings suggested that the risk of DVT in severe hemophilia A patients who underwent bilateral simultaneous TKA was relatively low. Thus, factor replacement therapy and TKA can be safely performed on hemophiliacs without pharmacological thromboprophylaxis.

TKA is an effective therapy to treat end-stage hemophilic arthritis, which could relieve pain, correct alignment, control bleeding and restore function [23, 24]. Simultaneous bilateral TKA is routinely considered since hemophilic arthritis usually damages both knees [1, 2]. In the general population, the first study of simultaneous bilateral TKA was reported by Hardaker et al. in 1978. This study compared the clinical outcomes in two groups of patients undergoing staged vs. simultaneous bilateral TKA [25]. Unfortunately, simultaneous bilateral TKA was reported to have a higher prevalence of complications, such as intraoperative blood loss and mortality, compared with staged unilateral TKA [10]. Conversely, in 2015, Mortazavi et al. assessed the safety and cost-effectiveness of simultaneous bilateral surgery in patients with hemophilia and they suggested that simultaneous bilateral TKA would not increase the rate of complications in patients with bilateral hemophilic knee arthropathy [26]. However, the latter neglected the incidence of DVT complications after bilateral simultaneous TKA.

It was previously reported that, without pharmacological thromboprophylaxis, the rate of subclinical VTE is as high as 50% in general patients who undergo orthopedic surgery [27, 28]. Nevertheless, the incidence of VTE in hemophiliacs was thought to be extremely low because of their defective coagulation system. Hermans et al. conducted a prospective study, which reported that the incidence of asymptomatic DVT in hemophiliacs after TKA or total hip arthroplasty was 10% (2 out of 20 procedures) [29]. According to a retrospective study of 25 TKAs performed in hemophilia patients in Japan, there were no cases of DVT detected on US performed 1 week postoperatively [12]. Considering the high bleeding risk in this population, pharmacological thromboprophylaxis was not applied. However, these studies mainly involved subjects undergoing unilateral TKA. Thus, it was unknown whether the prevalence of DVT increases in hemophiliacs undergoing bilateral simultaneous TKA.

Jenkins PV et al. reported that, in the general population, FVIII levels correlated with the risk of VTE events [30]. Theoretically, the administration of coagulation factor perioperatively rebalances the coagulation system, thus it might increase the hemophilia patients’ risk of DVT to the same level as the general population [31]. In an investigation of American hemophilia centers in 2009, 67% of surgeons considered that DVT prophylaxis should be performed in hemophiliacs who undergo joint replacement [32]. Possible thromboprophylaxis methods includes mechanical prophylaxis (intermittent pneumatic compression devices and graduated compression stockings) and pharmacological prophylaxis (the most commonly prescribed postoperative anticoagulant is low molecular weight heparin, but other oral medications include warfarin, direct oral anticoagulants, and aspirin). Mannucci et al. recommended that low molecular weight heparin is administered within 6–12 h following orthopedic surgery. However, this study also suggested that routine pharmacological prophylaxis has certain risks in hemophilia and better risk stratification is required to identify individuals who can benefit from anticoagulants [33].

Although pharmacological thromboprophylaxis was not used in the present study, no patient with severe hemophilia A was diagnosed with postoperative DVT after TKA. This can be explained by our institutional protocol which aims at FVIII levels around 100% on the day of operation; then, the dose of coagulation factor is gradually reduced. Furthermore, hemophilia patients usually undergo total joint arthroplasty at a relatively young age. Thus, the risk factors for DVT in these patients are fewer than for older patients without hemophilia who undergo total joint replacement. According to a prospective study, independent risk factors for DVT after TKA were age > 80 years and BMI > 35 kg/m^2^ using the Danish National Patient registry [34]. The majority of the patients in the present study had few risk factors for DVT and routine mechanical thromboprophylaxis may have had a preventive effect on DVT.

It remains controversial whether tourniquet use influences the incidence of DVT. Bin et al. suggested that the risk of DVT increased when the time of tourniquet use in TKA exceeded 60 min [35]. In our study, a tourniquet was utilized in all cases to reduce intraoperative blood loss. TKAs performed in our study with consistent tourniquet usage did not indicate an adverse effect on the incidence of DVT.

To the best of our knowledge, this is the first retrospective study to explore the risk of DVT in patients with severe hemophilia A treated with bilateral simultaneous TKA. There have always been controversies about whether hemophiliacs undergoing major orthopaedic surgeries need pharmacological anticoagulation. Our findings suggest that the risk of DVT in patients with haemophilia after TKA may be lower than that in the general population. This result may provide some guidance during clinical work in this area.

### Limitations

This study has several limitations. First, our study had a retrospective design. Confounding and bias are inherent in a retrospective study, despite the efforts made to reduce their impact on error. Second, due to the small number of patients enrolled and the very low incidence of VTE, inferential statistical analysis was not feasible. Third, most patients with hemophilic arthritis underwent TKA at a relatively young age, which led to lack of data in elderly hemophilia patients. Since the risk of DVT in this population might increase with age, our results might not be generalizable to elderly hemophilia patients undergoing TKA. Fourth, the follow-up period of our study was only two years, which is relatively narrow. Additional prospective studies with larger sample sizes and long follow-up periods are needed in the future.

## Conclusion

In this study, the risk of DVT in patients with severe hemophilia A after bilateral simultaneous TKA is found to be relatively low. Factor replacement therapy and TKA can be safely performed on this group of hemophiliacs without pharmacological thromboprophylaxis. As a result, routine thromboprophylaxis measures in this type of patients may not need to be the same as in the general population.

## Data Availability

The data that support the findings of this study are available from the corresponding author with proper reasons.
